# Viral reactivation in the lungs of patients with severe pneumonia is associated with increased mortality, a multicenter, retrospective study

**DOI:** 10.1002/jmv.28337

**Published:** 2022-11-29

**Authors:** Lingtong Huang, Xuan Zhang, Lisha Pang, Peng Sheng, Yanqiu Wang, Fan Yang, Huili Yu, Xiaohan Huang, Yue Zhu, Ning Zhang, Hongliu Cai, Lingling Tang, Xueling Fang

**Affiliations:** ^1^ Department of Critical Care Units, First Affiliated Hospital Zhejiang University School of Medicine Hangzhou China; ^2^ Department of Infectious Diseases, The First Affiliated Hospital Zhejiang University School of Medicine Hangzhou China; ^3^ Department of Critical Care Units Tongde Hospital of Zhejiang Province Hangzhou China; ^4^ Department of Critical Care Units The First Hospital of Jiaxing Jiaxing China; ^5^ Department of Critical Care Units Hangzhou Hospital of Traditional Chinese Medicine Hangzhou China; ^6^ Department of Nephrology, The First Affiliated Hospital Zhejiang University School of Medicine Hangzhou China; ^7^ Department of Emergency Medicine Lishui City People's Hospital Lishui China; ^8^ Department of Infectious Diseases, Shulan (Hangzhou) Hospital Zhejiang Shuren University of Shulan International Medical College Hangzhou China

**Keywords:** cytomegalovirus, Epstein‐Barr virus, herpes simplex virus, mNGS, reactivation

## Abstract

Viral reactivation is widespread in patients with severe pneumonia, yet the landscape of viral reactivation in the lungs is not well‐known. This study aims to assess the landscape and clinical features of viral reactivation in the early onset of severe pneumonia in ICU patients. The clinical data from 97 patients were collected retrospectively from the intensive care units of five teaching hospitals between June 2018 and July 2021. Metagenomic next‐generation sequencing (mNGS) of the bronchoalveolar lavage fluid (BALF) was performed at the onset of severe pneumonia. Cytomegalovirus (CMV), herpes simplex virus‐1 (HSV‐1), and Epstein‐Barr virus (EBV) were the most common reactivated viruses in the lower respiratory tract of patients with severe pneumonia. After adjusting for the risk of confounding and competition of age, sex, sequential organ failure assessment, acute physiology chronic health assessment II and immunosuppression status, viral reactivation resulted in an overall 2.052‐fold increase in 28‐day all‐cause mortality (95% CI: 1.004–4.194). This study showed that CMV, HSV‐1, and EBV were the most common reactivated viruses in the lungs of patients with severe pneumonia. The existence of viral reactivations was associated with an increased risk of mortality. The simultaneous reactivation of multiple viruses needs to be considered in the design of clinical trials.

## 1 INTRODUCTION

Severe pneumonia is one of the leading causes of death in the intensive care unit (ICU) and is often accompanied by the reactivation of cytomegalovirus (CMV),^1^ herpes simplex virus (HSV),^2^ and Epstein‐Barr virus (EBV) during the disease treatment. CMV or EBV reactivation was always assessed using serum samples,^3^, ^4^, ^5^ while HSV‐1 reactivation was detected through bronchoalveolar lavage fluid (BALF).^6^ There has also been a recent widespread viral reactivation in patients with severe pneumonia who have been transferred to the ICU due to the severe Coronavirus Disease 2019 (COVID‐19).^7^, ^8^, ^9^ Reactivation of CMV, HSV‐1, and EBV is associated with poor prognosis.^10^, ^11^, ^12^

Metagenomic next‐generation sequencing (mNGS) is not routinely used for unbiasedly detecting pathogenic microorganisms.^13^, ^14^ In this study, the BALF of ICU patients with severe pneumonia was analyzed by mNGS. The results indicated that CMV, HSV‐1, and EBV were the most commonly reactivated viruses in the lungs of these patients. Currently, few studies have evaluated the reactivation of these viruses in the lower respiratory tract simultaneously and their contribution to the mortality of patients with severe pneumonia in the ICU. This study aimed to assess the clinical characteristics of this research gap in patients with severe pneumonia.

## 2 METHODS

### 2.1 Study design and patient population

In this multicenter retrospective study, mNGS detection was performed on the BALF of 97 patients with severe pneumonia admitted to the ICU in the following five teaching hospitals: the First Affiliated Hospital of Zhejiang University School of Medicine, Tongde Hospital of Zhejiang Province, the First Hospital of Jiaxing, Hangzhou Hospital of Traditional Chinese Medicine, and Lishui City People's Hospital. The inclusion criteria of patients were as follows: aged over 18‐year‐old and transferred to the ICU and diagnosed with severe pneumonia. Given that these patients have a clear primary disease, and none of them were diagnosed with HSV‐1 pneumonia, CMV pneumonia or EBV pneumonia, the definition of reactivation was the presence of CMV or HSV‐1, or EBV in the lung detected by mNGS. The viral load in alveolar lavage fluid was affected by balf acquisition and different lung segments from which samples were obtained, so we divide these patients into those with herpesviruses detected (reactivation) and those without herpesviruses detected (nonreactivation) without discussing the specific abundance. The immunosuppressive status was defined as follows: (1) Peripheral blood neutropenia, less than 0.5 × 10^9/L for 10 days after admission; (2) Taking immunosuppressive drugs within 30 days before the mNGS test, such as tacrolimus, cyclosporine, mycophenolate mofetil, or the use of monoclonal antibodies such as rituximab; (3) History of AIDS, hematological tumors, or transplantation.

### 2.2 Data collection and mNGS assay of BALF

For all included patients, the demographic data such as gender, age, days of ICU stay, days of mechanical ventilation, days from admission to mNGS testing, community‐acquired pneumonia, immunosuppressive status, and patient outcomes on day 28 were recorded. To assess the disease severity, the acute physiology and chronic health evaluation II (APACHE II) and sequential organ failure assessment (SOFA) were calculated when the patients were admitted to the ICU and on the day of mNGS testing.

Low‐speed centrifugation (1500*g* for 20 min) was performed to remove human cells in the samples. Samples were then homogenized using bead beating, followed by DNA extraction using the IngeniGen DNA Extraction Kit (IngeniGen XMK Biotechnologies Inc). The DNA libraries were prepared using the IngeniGen DNA Library Prep Kit following the manufacturer's protocols. Briefly, the DNA was fragmented, and the Illumina‐compatible adapters were simultaneously added to the fragmented DNA by a tagmented enzyme. The library was purified using magnetic beads and then amplified after 15 PCR cycles. Samples were then homogenized using bead beating, followed by RNA extraction using the IngeniGen RNA Extraction Kit (IngeniGen XMK Biotechnologies, Inc). The RNA libraries were constructed using the IngeniGen XMKbio RNA‐seq Library Prep Kit (IngeniGen XMK Biotechnologies, Inc). Briefly, DNase was used to remove residual human DNA, and RNA was fragmented, followed by double‐strand cDNA synthesis, end‐repair, dA‐tailing, and adapter ligation. Sequencing was performed on Illumina MiniSeq (Illumina) using 2 × 75 bp chemistry for 78 samples and Illumina NextSeq (Illumina) using 1 × 100 bp chemistry for 19 samples. A negative control was included in each run to detect background contaminants, and internal control was added to each sample to monitor the entire process. Data analysis was performed as follows: First, the fastp software (https://github.com/OpenGene/fastp) was used to filter raw sequencing data to remove adapters and low‐quality sequences, and then the bowtie2 software (http://bowtie-bio.sourceforge.net/bowtie2/index.shtml) aligned the sequences to the human reference genome (GRCh38) to remove host sequences.^15^, ^16^ The processing results of reads are provided in the Supporting Information. The remaining sequences were classified and annotated using the Kraken2 (https://ccb.jhu.edu/software/kraken2/) software, and the results were visualized using the pavian tool (http://ccb.jhu.edu/software/pavian/).^17^ Pavian outputs from Kraken2 results are provided in the Supporting Information. The raw data of mNGS is available from the Genome Sequence Archive for Human (HRA002995).

### 2.3 Statistical analysis

The statistical analysis was performed using SPSS statistical package version 26. Data were first summarized using standard descriptive statistics. Mann–Whitney *U* and *χ* ^2^ tests were used to analyze the clinical characteristics. Cox proportional hazards models, hazard ratios (HR), and confidence intervals (CI) were estimated to identify predictors of mortality or survival in‐hospital. The Kaplan–Meier analysis was performed to determine patient survival in the nonreactivation and reactivation groups. Unadjusted HR was reported, and *p* < 0.05 were considered statistically significant. Figures were prepared using GraphPad Prism 8 or R 4.1.1.

## 3 RESULTS

### 3.1 mNGS results

**Figure 1:**
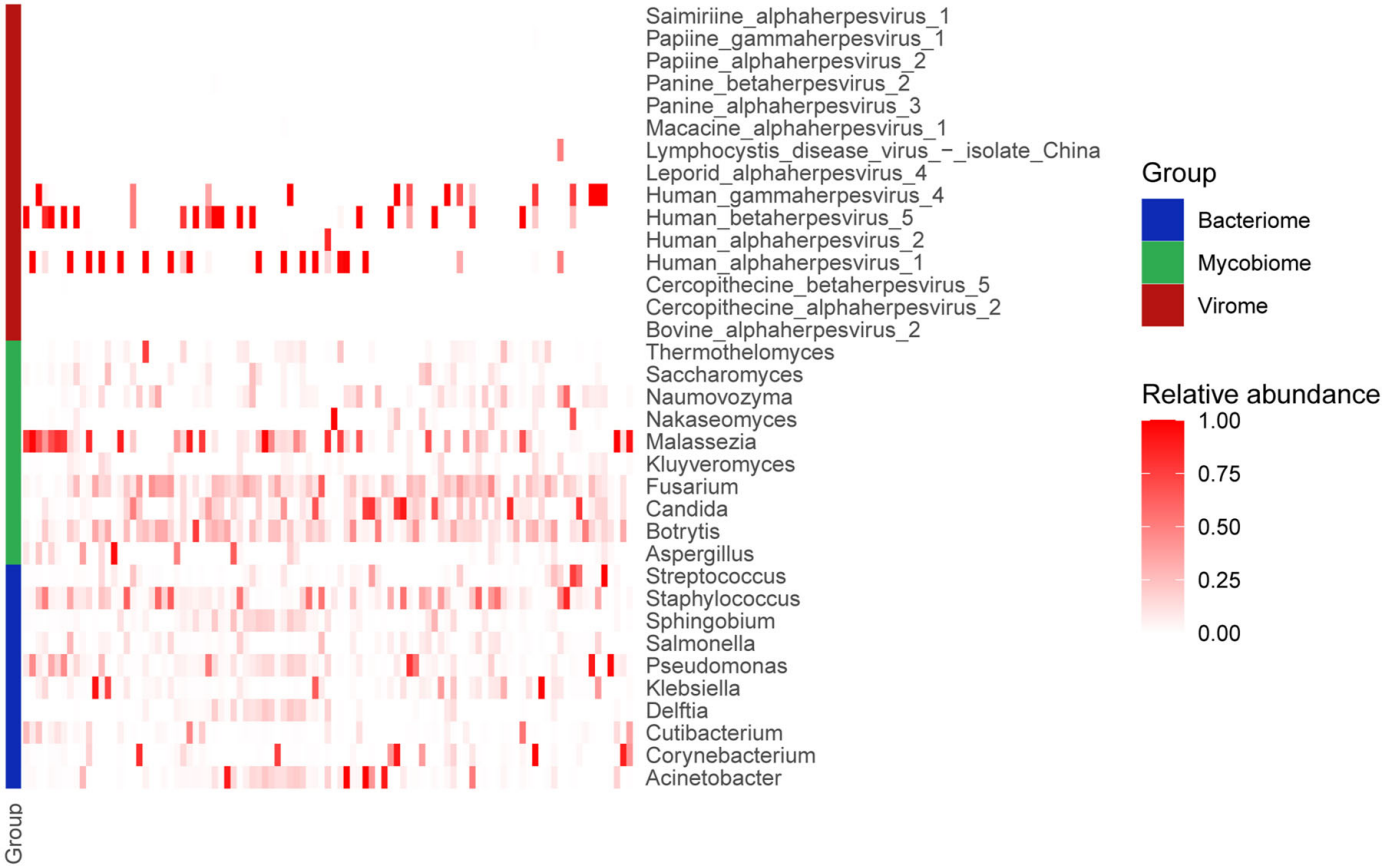
Overview of the mNGS analysis of Bacteriome, Mycobiome and Virome. Heatmap illustrating the relative abundance of key taxa within bacterial, fungal and viral communities. Each column represents a patient.

In the lungs of patients with severe pneumonia, mycobiome was composed of *Malassezia, Candida, Botrytis, Thermothermomyces, Saccharomyces, Naumovozyma, Nakaseomyces, Kluyveromyces, Fusarium, Aspergillus*, and so forth (Figure 1). The 10 most frequently detected bacteria were *Klebsiella, Pseudomonas, Acinetobacter, Streptococcus, Staphylococcus, Sphingobium, Salmonella, Delftia, Cutibacterium, and Corynebacterium* (Figure 1). We then compared mycobiome and bacteriome of patients who survived or died during 28‐day which showed the higher relative abundance of *Kluyveromyces* (*p* < 0.05) and *Candida* (*p* < 0.05) in nonsurvival group (Supporting Information). Unlike the widespread presence of bacteriome and mycobiome in the lower respiratory tract, only some viruses appear in the lower respiratory tract of critically ill patients with pneumonia, with CMV, HSV‐1, and EBV being the most frequent (Figure 1).

### 3.2 Patient characteristics

In this study, the participants were classified based on the presence of CMV, HSV‐1, and EBV. The nonreactivation and viral reactivation groups included 51 and 46 patients, respectively (Table 1). The two groups of patients were not significantly different in terms of age, gender, length of hospital stay, length of stay in ICU, days of ventilator use, or time from admission to mNGS assay.

**Table 1:** Clinical characteristics

	Total (*n* = 97)	Nonreactivation (*n* = 51)	Reactivation (*n* = 46)	*p* Value
Age, year	65 (53–73)	64 (53–74)	66 (52.5–71.5)	0.991
Male gender (%)	57 (58.8)	33 (64.7)	24 (52.2)	0.224
hospital stay, day	26 (14.5–47)	27 (16–53)	24.5 (13–43.5)	0.463
ICU stay, day	16 (10–32.5)	16 (10–30)	13.5 (7.5–39.25)	0.58
CAP (%)	60 (61.9)	33 (64.7)	27 (58.7)	0.676
Immunosuppression (%)	36 (37.1)	12 (23.5)	24 (52.2)	**0.006**
Mechanical ventilation, day	12 (6–26)	12 (6–27)	12 (5–24.25)	0.641
SOFA score at transfer to ICU	8 (4.5–12)	8 (4–12)	7.5 (5–12)	0.848
APACHE II score at transfer to ICU	18 (13–25.5)	19 (13–24)	17.5 (13–27.25)	0.828
NGS testing of Balf				
Days after admission to hospital, day	3 (2–11)	4 (2–9)	3 (1–14)	0.675
SOFA score at testing	8 (5–12)	8 (4–12)	8 (6–12)	0.651
APACHE II score at testing	19 (15–25.5)	19 (15–25)	20 (16–28.5)	0.296
28‐day mortality (%)	41 (42.3)	17 (33.3)	24 (52.2)	0.06

*Note*: Mann–Whitney *U*‐test and the *χ* ^2^ test were used to analyze the clinical characteristics, *p* < 0.05 were considered statistically significant and shown in bold.

Abbreviations: APACHE II, acute physiology and chronic health evaluation II; CAP, community‐acquired pneumonia; SOFA, sequential organ failure.

There was no statistically significant difference between the SOFA and APACHE II at admission or at mNGS testing, which suggested that the two groups of patients had similar severity. However, 24 of the patients (52.2%) in the reactivation group had an immunosuppressed state, which is significantly higher than 12 of the patients (23.5%) in the nonreactivation group (*p* = 0.006).

### 3.3 Twenty‐eight‐day all‐cause mortality

**Figure 2:**
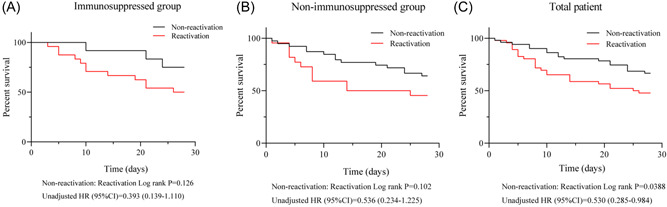
(A–C) Kaplan–Meier curve of immunosuppressed (A) nonimmunosuppressed (B) or total patient (C) at 28 days after mNGS detection

Survival curves were plotted to observe the survival status of the patients in several groups (Figure 2A–C). The Kaplan–Meier curves of the total patients indicate significantly lower mortality rates in the non‐reactivation group with unadjusted influence versus the reactivation group (unadjusted HR, 0.53; 95% CI: 0.285–0.984; *p* = 0.0388). Besides, the Kaplan–Meier curves of the immunosuppressed group (Nonreactivation: Reactivation, unadjusted HR, 0.393; 95% CI: 0.139–1.110) or nonimmunosuppressed group (Nonreactivation: Reactivation, unadjusted HR, 0.536; 95% CI: 0.234–1.225) showed a similar trend. Viral reactivation was associated with a 28‐day increase in all‐cause mortality. However, due to the small sample size of the subgroup, no statistical significance was obtained. After a prior adjustment for age, sex, immunosuppressive status, and APACHE II and SOFA scores, the association remained significant. The viral reactivation in the lower respiratory tract was identified as an independent risk factor for death in patients with severe pneumonia (adjusted HR, 2.052; 95% CI: 1.004–4.194; *p* < 0.05) (Table 2).

**Table 2:** Multivariable analysis

	HR (95% CI)	*p* Value
Age	0.994 (0.974–1.015)	0.578
Male gender	0.579 (0.286–1.172)	0.129
Immunosuppression state	1.115 (0.549–2.266)	0.764
SOFA score at testing	1.159 (1.069–1.257)	**<0.001**
APACHE II score at testing	1.031 (0.986–1.078)	0.183
Viral reactivation state	2.052 (1.004–4.194)	**0.049**

*Note*: The results of multivariable analyses for 28 day all‐cause mortality with the Cox regression model, *p* < 0.05 were considered statistically significant and shown in bold.

Abbreviations: APACHE II, acute physiology and chronic health evaluation II; SOFA, sequential organ failure.

## 4 DISCUSSION

In this study, we performed mNGS assays on BALF and showed that the virome in the lower respiratory tract of patients with severe pneumonia was composed of CMV, HSV‐1, and EBV. There are few studies on viral reactivation in critically ill pneumonia patients in the ICU. Recent studies on HSV‐1 and CMV used blood samples to detect viral reactivation. However, blood samples are not a reliable tool for assessing viral reactivation in the lungs. In addition, most studies failed to simultaneously assess HSV‐1, CMV, EBV reactivation, and clinical characteristics of patients, similar to previous studies that utilized only a single RT‐PCR for detection.^5^, ^18^ Our study revealed that CMV, HSV‐1, and EBV reactivations were associated with increased 28‐day all‐cause mortality in patients. However, we did not perform qPCR validation, which made it impossible for us to assess the accurate load of CMV, HSV‐1, and EBV in the lungs of patients. Studies that evaluate HSV‐1, CMV, or EBV reactivation alone may be limited by the absence of adjustments for confounding factors due to the small sample size. Accordingly, approximately 400 samples are needed to accurately assess how the reactivation of these three viruses affects patient mortality. we compared the mycobiome and bacteriome composition of survival patients and nonsurvival patients. Among the fungi and 10 bacteria with the highest abundance, only *Kluyveromyces* and *Candida* showed statistical differences in survival and nonsurvival group. In view of the fact that the two fungi detected are not pathogenic, we believe that this result have no clinical significance. Meanwhile, due to the small sample size of this study, we did not analyze the effect of other species detected by mNGS.

Approximately 47.4% of the patients had a CMV, HSV, or EBV reactivation after admission. The incidence of herpes viremia increased proportionally with the length of hospital stay, and on day 28, almost all the patients were positive for herpes viremia.^5^ As part of this study, mNGS was performed only at the beginning of the illness, which may underestimate the increased incidence of viral reactivation with a longer disease duration. Considering that high frequency of simultaneous reactivation of CMV, HSV‐1, and EBV, few studies on the effect of ganciclovir alone to prevent CMV reactivation on patient outcomes have achieved statistical significance.^18^ A combination of CMV, HSV‐1, and EBV reactivation prevention may reduce the mortality in patients with severe pneumonia in the ICU. Randomized controlled trials are thus needed for confirmation.

## 5 CONCLUSION

This study showed that CMV, HSV‐1, and EBV were the most commonly reactivated viruses in the lungs of patients with severe pneumonia. The existence of viral reactivation was associated with an increased risk of mortality. This simultaneous reactivation of multiple viruses needs to be considered in the design of clinical trials.

## AUTHOR CONTRIBUTIONS

Xueling Fang, Lingtong Huang, Lingling Tang designed the clinical trial. Lingtong Huang, Lisha Pang, Hongliu Cai, Peng Sheng, Yanqiu Wang, Ning Zhang, Huili Yu, Xiaohan Huang, Yue Zhu, and Fan Yang collected clinical data from each center. Lingtong Huang, Xuan Zhang performed statistical analysis of the data. Xueling Fang and Lingling Tang drafted the manuscript, prepared the figures and critically reviewed the final manuscript. All authors contributed to the article and approved the submitted version.

## CONFLICT OF INTEREST

The authors declare no conflict of interest.

## ETHICS STATEMENT

The ethics committees of the five participating institutions including the First Affiliated Hospital of Zhejiang University School of Medicine, Tongde Hospital of Zhejiang Province, the First Hospital of Jiaxing, Hangzhou Hospital of Traditional Chinese Medicine and Lishui City People's Hospital approved the study protocol. Since the research involved retrospective data, written informed consent was waived off.

## Supporting information

Supplementary information.

Supplementary information.

## Data Availability

The data sets used and/or analyzed during the current study are available from the corresponding author on reasonable request. The raw data of mNGS was available at the Genome Sequence Archive for Human (HRA002995).
