# Supplementary material for: Viral reactivation in the lungs of patients with severe pneumonia is associated with increased mortality, a multicenter, retrospective study
Source: J Med Virol. 2022 Nov 29;95(1):e28337. doi: 10.1002/jmv.28337 (PMC10099828; doi:10.1002/jmv.28337)
Supplement: Supplementary file 2 — Supplementary information. [file JMV-95-0-s002.docx]

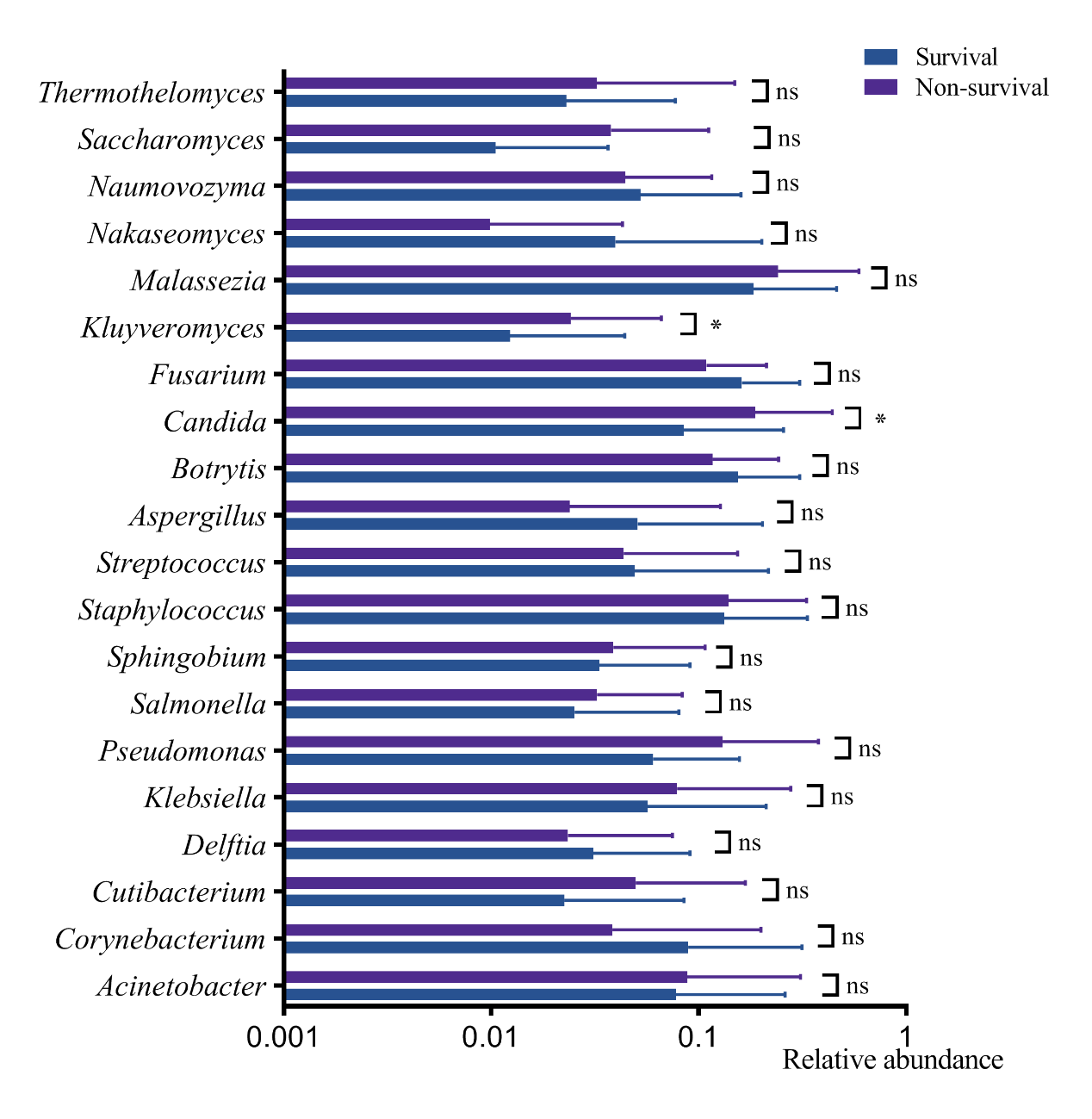


Figure legend

The difference between the ten fungi and ten bacteria with the highest abundance in the 28-day survival group and non-survival group. Mann–Whitney U test were used to analyze the relative abundance, *p-*values < 0.05 were considered statistically significant.
